# Innovative biological approaches for monitoring and improving water quality

**DOI:** 10.3389/fmicb.2015.00826

**Published:** 2015-08-12

**Authors:** Sanja Aracic, Sam Manna, Steve Petrovski, Jennifer L. Wiltshire, Gülay Mann, Ashley E. Franks

**Affiliations:** ^1^Applied and Environmental Microbiology Laboratory, Department of Physiology, Anatomy and Microbiology, La Trobe University, Melbourne, VIC, Australia; ^2^Land Division, Defence Science and Technology Organisation, Melbourne, VIC, Australia

**Keywords:** synthetic biology, bioremediation, electromicrobiology, phage-therapy, water quality

## Abstract

Water quality is largely influenced by the abundance and diversity of indigenous microbes present within an aquatic environment. Physical, chemical and biological contaminants from anthropogenic activities can accumulate in aquatic systems causing detrimental ecological consequences. Approaches exploiting microbial processes are now being utilized for the detection, and removal or reduction of contaminants. Contaminants can be identified and quantified *in situ* using microbial whole-cell biosensors, negating the need for water samples to be tested off-site. Similarly, the innate biodegradative processes can be enhanced through manipulation of the composition and/or function of the indigenous microbial communities present within the contaminated environments. Biological contaminants, such as detrimental/pathogenic bacteria, can be specifically targeted and reduced in number using bacteriophages. This mini-review discusses the potential application of whole-cell microbial biosensors for the detection of contaminants, the exploitation of microbial biodegradative processes for environmental restoration and the manipulation of microbial communities using phages.

## Introduction

Anthropogenic activities, such as manufacturing, mining and farming, have led to an increase of a wide range of chemical (organic and inorganic) and biological (e.g., bacteria, yeast, fungi, and algae) contaminants in aquatic environments (Carpenter et al., 2011). Contamination of lakes, rivers, oceans, reservoirs, and groundwater affects not only the organisms living within these bodies of water but can also impact the entire biosphere. A major threat to aquatic ecosystems worldwide is eutrophication, the over-enrichment of water with nutrients or organic matter (Woodward et al., 2012). High concentrations of nitrogenous compounds and phosphates result in the formation of algal blooms which negatively impact water quality and ecosystems (Grizzetti et al., 2012). Inorganic contaminants, in particular heavy metals, are also a prominent environmental concern because they are not biodegradable and can accumulate in living organisms (Fu and Wang, 2011). The toxicity of heavy metals depends not only on the particular element but also its chemical speciation and oxidation state. The threat posed to higher organisms as a consequence of contaminated water can be reduced by microbial degradation of organics and re-speciation of heavy metals into less toxic forms (Kot and Namiesńik, 2000).

The current approaches for removal of contaminants include sedimentation, membrane filtration, coagulation-flocculation, adsorption, chemical precipitation and ion-exchange (Otte and Jacob, 2006; Fu and Wang, 2011). These processes depend on existing infrastructure and can be impractical to implement in developing countries and remote areas. As a result, there is an increasing need to detect and treat contaminated water *in situ* using sustainable approaches that are inexpensive and environmentally-friendly. Researchers are currently investigating the use of native biota for the detection and degradation or reduction of contaminants as a cheaper and sustainable alternative. This mini-review addresses the potential application of biological approaches, and their limitations, as complementary methods to chemical techniques for the detection of contaminants and treatment of contaminated water.

## Monitoring of Water Quality

### Detection of Contaminants Using Naturally-existing Whole-cell Microbial Biosensors

Environmental and microbiological research has driven the growing interest in real-time monitoring of water quality using whole-cell microbial biosensors. Yeast, algae and bacterial whole-cell biosensors have been applied to domestic wastewater and natural waters to detect phenols, non-ionic surfactants, pesticides, heavy metals and effluents from the chemical industry (Girotti et al., 2008). Microbial whole-cell biosensors produce a measurable signal enabling detection and quantification of contaminants (Lagarde and Jaffrezic-Renault, 2011). Growth characteristics, enzymatic activity or other measureable outputs can be monitored in response to the presence of specific contaminants. Given their ubiquity in aquatic systems algae have been utilized as bioreporters that are capable of detecting contaminants and nutrient fluxes in water. The abundance of specific benthic algae (16 of 21 species tested) directly correlated with the total phosphorus present, providing information on *in situ* levels (Rott and Schneider, 2014). The morphological responses of cyanobacteria to specific nutrients also provides information on nutrient levels, in the absence of nitrogen these organisms form an abundance of nitrogen-fixing heterocysts, whereas in the absence of phosphorus they produce elongated filaments (Whitton and Potts, 2007).

**FIGURE 1:**
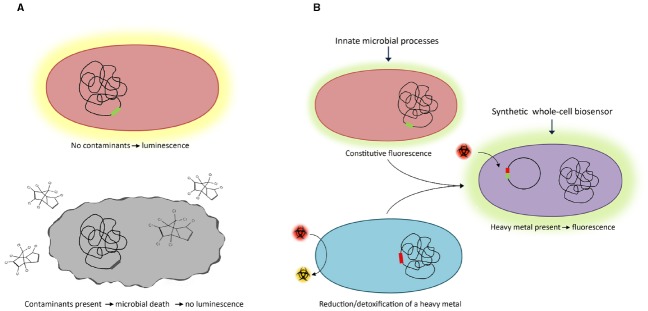
**Monitoring water quality using (A) naturally-existing and (B) genetically-synthesized microbial biosensors**.

Some microorganisms possess innate characteristics, such as luminescence or the ability to generate electrical current, which can be utilized to measure metabolic response to environmental contaminants (Figure 1A; Daunert et al., 2000). Luminescence produced by the marine bacterium *Vibrio fischeri* has been exploited for the detection of phenols in water (Stolper et al., 2008). The presence of phenols in water results in a quantifiable reduction of luminescence of the microorganism (a 90% reduction of luminescence was observed in the presence of 100 mg L^–1^ 3,4-dichlorophenol) (Stolper et al., 2008). While *Vibrio* uses luminescence as a phenotypic indicator, there are other naturally existing biosensors that use non-luminescent based strategies to report the presence or absence of contaminants in water.

Current production in microbial fuel cell systems, a measure of electron flow from central metabolism, is a direct measure of metabolic activity and can be used to monitor changes in metabolic activity over time (Aracic et al., 2014). This approach has been utilized for *in situ* monitoring of the metabolic activity of complex microbial communities in a variety of subsurface anoxic environments (Williams et al., 2009). The indigenous microbial population may utilize many contaminants as electron donors and cause an increase in microbial metabolism. Wastewater contains a large volume of organic compounds that can stimulate microbial growth. Metabolic activity, as measured as current production in microbial fuel cells, has been shown to be capable of providing real-time monitoring of organic effluents in relation to their chemical oxygen demand (Di Lorenzo et al., 2009). Likewise, a simple anode-resistor-cathode device can monitor *in situ* rates of anoxic subsurface microbial activity providing continuous metabolic rates in response to the presence of organic contaminants (Wardman et al., 2014). The integration of nanomaterials (gold particles, magnetic beads and carbon nanotubes) as well as electron mediators in electrochemical biosensors has resulted in improved limits of detection of numerous water pollutants (Lagarde and Jaffrezic-Renault, 2011). Immobilization of electroactive *Pseudomonas putida* cells on electrodes using carbon nanotubes resulted in an 80-fold increase in sensitivity and 2.8-fold increase in response time to trichloroethylene (Hnaien et al., 2011). In more recent years biosensor research has moved from naturally existing whole-cell biosensors to synthetically-derived microbial biosensors to optimize the detection of contaminants. Advances in synthetic biology have allowed the stability, specificity and sensitivity of whole-cell biosensors to be improved.

### Detection of Contaminants Using Synthetically-derived Microbial Biosensors

Synthetic biology is now allowing the systematic design of whole-cell biosensors. Typically, a reporter gene is placed under the control of a promoter that is transcriptionally active in the presence of a specific contaminant (Figure 1B). Numerous regulatory elements (promoters and their cognate transcriptional regulators) have been identified which respond to specific organic contaminants and heavy metals found in contaminated water such as arsenic, cadmium and mercury (Bereza-Malcolm et al., 2014). The regulatory elements control the transcription of reporter genes whose expression produces a detectable and quantifiable fluorescent, luminescent or electrochemical signal. Synthetically-derived microbial biosensors are often created using common laboratory strains of *Escherichia coli*. While these systems are functional in *E. coli*, a significant challenge is encountered in the real-world application of these biosensors for the detection of contaminants in aquatic settings. This is because *E. coli* lacks many of the physiological characteristics that are required for its survival and proliferation in these niche environments. As a consequence, biosensors are being developed using microbes that inhabit the aquatic environment of interest. Cyanobacteria, which inhabit marine and freshwater environments, have been engineered to detect and provide a measurable signal in response to a range of contaminants. Genetically-modified cyanobacteria have been used as *in situ* bioindicators for excess nutrients, organic contaminants and heavy metals in water (Gillor et al., 2010; Mateo et al., 2015). An extensive range of strains have been engineered as general ecotoxicity, nitrogen, ammonium, nitrate, nitrite, phosphorus and heavy metals reporters using luciferase in a light-on or lights off response (see Mateo et al., 2015, for a recent extensive review).

The use of electroactive microorganisms as whole-cell biosensors allows the integration of microbial outputs into electrochemical signals. The ability of these organisms to transfer electrons to and from electrodes has been extensively studied for numerous biotechnological processes including their potential use in autonomous sensing devices (Rosenbaum and Franks, 2014). A *Shewanella oneidensis* biosensor has been constructed using outer membrane cytochrome complexes capable of electron transfer under the control of a promoter responsive to arabinose. The concentration-dependent detection of arabinose was linked to the expression of cytochromes involved in direct extracellular electron transfer (Golitsch et al., 2013). Proteins involved in electron transfer (flavins, shuttles, and cytochromes) produce specific electrochemical signals detectable through electrochemical techniques (Golitsch et al., 2013). The required instrumentation for electrochemical detection can be miniaturized, providing portable analytical devices for simple *in situ* measurements (Badihi-Mossberg et al., 2007).

Whole-cell biosensors can detect contaminants with sensitivities comparable to those of conventional chemical methods and/or optical bioassays (Lagarde and Jaffrezic-Renault, 2011). Therefore they could serve as complementary techniques to conventional chemical methods currently used to monitor levels of contaminants by the Environmental Protection Agency and World Health Organization. Furthermore, microbial biosensors measure the bioavailability of the contaminants without the need for further *in vitro* bioassays. Nevertheless, several limitations still remain in the application of biosensors including the variability and complexity of flowing water, the wide range of concentrations of contaminants, as well as the sensitivity, specificity and robustness of the biosensors.

## Improving Water Quality

### Enhanced Removal of Organic Contaminants and Heavy Metals Via Exploitation of Macrophyte-associated Microbial Communities

**FIGURE 2:**
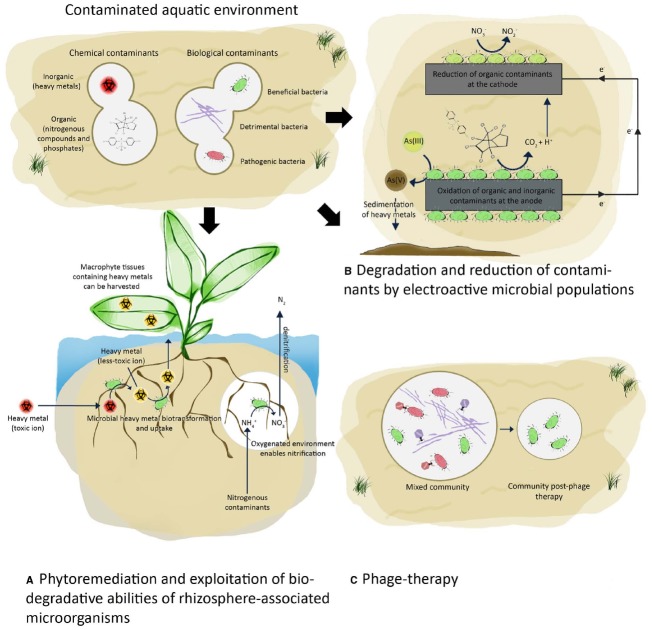
**Improving water quality of aquatic environments using biological approaches.** Bioremediation of contaminated water by **(A,B)** exploitation of microbial biodegradative abilities and **(C)** manipulation of microbial communities using phages.

Excessive nitrogen and phosphorus in water causes proliferation of algae resulting in reduced oxygen availability, food resources and habitats that fish and other aquatic life need to survive. Algal blooms are also harmful because they produce elevated toxins and can result in increased bacterial growth. The reversal of eutrophication (oligotrophication) and whether reduction of nitrogen, phosphorus or both is the most cost-effective approach to improve water quality has been extensively reviewed (Grizzetti et al., 2012; Schindler, 2012; Woodward et al., 2012). Nevertheless, specific processes are known to be crucial for the removal of these elements. Nitrification, performed mainly by bacteria from the *Nitrobacteraceae* family, is a process which reduces nitrogenous compounds from the environment (Hagopian and Riley, 1998). Under anoxic conditions the efficiency of nitrification is restricted (Yoo et al., 1999). This rate-limiting step can be enhanced fourfold via the use of vascular plants that live in or near water (macrophytes) which create oxygenated microenvironments in the rhizosphere and promote nitrification (Soana and Bartoli, 2014). The presence of contaminant-tolerant oxygen-releasing plants can stimulate nitrification by increasing microbial activity in the rhizosphere of otherwise anoxic sediments (Figure 2A). Artificial floating islands (i.e., soil-less structures constructed with floating macrophytes) have been applied in the treatment of stormwater, household effluent and industrial waste (Yeh et al., 2015). Numerous in-field studies have demonstrated that macrophyte-microbe interactions are responsible for the removal of organic matter in a range of polluted water environments (Yeh et al., 2015). For example, a floating bed of perennial grasses removed 60% of the total nitrogen and 56% of the total phosphorus, of which only 2.8 and 4.5%, respectively, were due to plant uptake (Li et al., 2012). The majority of the removal was due to the plant–microbe interactions, which were enhanced by the growing plants. The introduction of plants also resulted in 50% reduction of total petroleum hydrocarbons (from 1700 to 871 mg L^–1^). This approach is an environmentally-friendly strategy for removing excess nutrients and organic pollutants.

In addition to degrading excess nutrients and organic pollutants, macrophyte-associated microorganisms also facilitate heavy metal uptake, a crucial step in the phytoremediation process (Jing et al., 2007). This bioremediation strategy for treating contaminated water has an added benefit because harvested macrophytes can be processed into biomaterials such as biogas (Ahalya et al., 2003) and animal feed (Li et al., 2012). The protein and fiber content of macrophytes meet the national feed thresholds and the content of toxic heavy metal ions is below the critical levels for animal feeds (Li et al., 2012). The removal of macrophytes impedes nitrification and other microbial processes requiring oxygenated environments, however, if not removed, the decaying macrophytes can also serve as a source of pollution (Xie et al., 2013). An equilibrium between removing heavy metals while promoting the growth of a diverse range of microbes that degrade complex organic compounds is required for the process to be successful on a large scale. Further elucidation of the interplay between the chemical and biological processes that occur between macrophytes and microbes will lead to improved water bioremediation processes. The influence of environmental changes on these interactions as well as biotic and abiotic processes that compete for oxygen and nutrients, limiting their availability to the important microorganisms needs to be further explored.

### Enhanced Removal of Organic Contaminants and Reduction of Heavy Metals Via Exploitation of Microbial Communities: Biodegradative and Electroactive

Bioremediation of contaminated water can occur through innate processes performed by microbes, which involve the utilization of contaminants as a nutrient or energy source (Singh et al., 2014). Biodegradative microbes may be indigenous to the site or exogenous species used for bioaugmentation (Dixit et al., 2015). The growth and biodegradative abilities of these microbes can be enhanced through the addition of certain nutrients which promote proliferation of beneficial bacterial species or addition of terminal electron acceptors/donors. The stimulated microbial communities are able to convert organic and inorganic contaminants into non-hazardous or less hazardous forms through oxidation or reduction (Dixit et al., 2015). Often a consortium of microorganisms, including bacteria, yeast and fungi, performs these processes sequentially. The genetic architecture of these microbial communities allows biodegradation, biotransformation, biosorption and bioaccumulation of contaminants to occur in unison. These innate abilities can be further enhanced through genetic modification of regulatory and/or metabolic genes (Dixit et al., 2015). Heavy-metal-tolerant bacteria containing the arsenic(III) *S*-adenosylmethionine methyltransferase (ArsM) are able to methylate toxic inorganic arsenic(III) to the less toxic arsenic(V) (Chen et al., 2014). Arsenic volatilization can be improved ninefold by genetically engineering strains to overexpress ArsM. Although this approach has not yet been applied to treat arsenic contaminated water, the laboratory-scale study conducted by Chen et al. (2014) provides promising data to tackle arsenic contaminated environments.

Metagenomic studies are providing information about the composition and the genetic potential of microbial communities involved in the removal of excess elements and toxic contaminants (Bai et al., 2014). The presence of genes involved in xenobiotic degradation would indicate the presence of these contaminants in the aquatic environment. However, metatranscriptomics or gene expression studies are necessary to confirm the link between the abundance of degradation genes and biodegradation rates. RT-qPCR studies revealed that expression of hydrocarbon degrading genes of *Pseudomonas* and *Rhodococcus* species was 1000-fold higher in contaminated soil environments and were low or undetected in uncontaminated samples (Yergeau et al., 2012). Similarly, Yu and Zhang (2012) analyzed the presence and expression of genes involved in nitrification, denitrification, ammonification, and nitrogen fixation processes using metagenomic and metatranscriptomic analyses of microbial communities in wastewater (Yu and Zhang, 2012). This study revealed that although denitrification genes were most abundant (78.6%) of the four processes and nitrification genes were the least abundant (0.9%), the ratio of cDNA to DNA was highest for the nitrification process (0.18 and only 0.03 for denitrification). Therefore gene expression studies are essential in determining the potential for bioremediation of contaminated aquatic environments.

Electroactive microorganisms can use heavy metals and trace elements as terminal electron acceptors or reduce them through detoxification mechanisms. Dissimilatory metal reducing bacteria, such as species of *Geobacter*, utilize insoluble iron(III) or manganese(IV) as final electron acceptors (Lovley and Phillips, 1988). *Geobacter* spp. can also reduce soluble uranium(VI) to insoluble uranium(IV), thus precipitating the ions out of the water table (Lovley and Phillips, 1992; Anderson et al., 2003; Gregory and Lovley, 2005). Electron donors and acceptors can become limiting factors during degradation in anoxic environments. The innate capacity of these microorganisms to reduce/oxidize heavy metal ions and other contaminants can be improved for optimal removal of chemical contaminants using solid surfaces such as anodes as electron acceptors and cathodes as electron donors (Figure 2B; Du et al., 2007; Zhang et al., 2010). The transfer of electrons from microorganisms to the solid surface of an electrode enables the catabolism of chemical contaminants to take place at a significantly greater efficiency. For example the presence of an electrode as an electron acceptor instead of iron(III) oxide increased the speed of toluene oxidation 100-fold in petroleum-contaminated sediments (Zhang et al., 2010). While the catabolism of a wide range of organic substrates found in wastewater has been demonstrated (Daunert et al., 2000; Aulenta et al., 2009), significant challenges remain in the implementation of this system on a large scale. A better understanding of the mechanisms involved in electron transfer between mixed communities and electrode surfaces are needed to optimize the electron transfer *in situ* which would consequently increase the degradation efficiency (Aracic et al., 2014).

### Controlling Abundance of Biological Contaminants Using Phages

Phage-therapy is a process that allows the removal of a select subset of microbial populations within a water-associated community (Figure 2C). Excessive proliferation of filamentous bacteria in activated sludge systems is a major concern, affecting the crucial separation of the solid and liquid phases in settling tanks (De Los Reyes, 2010; Wanner et al., 2010). These processes are essential for successful wastewater treatment and are referred to as sludge bulking and foaming. Sludge bulking occurs when filamentous bacteria form aggregates (flocs) which can extend and interact with adjacent flocs thus disrupting the settleability of the solid particles. The proliferation of hydrophobic bacteria in aeration tanks consisting of surfactants results in the formation of stable foams (De Los Reyes, 2010; Petrovski et al., 2011a). In the absence of surfactants, hydrophobic cells form a stable scum layer on the top of the aeration tank (Petrovski et al., 2011a). The organisms responsible for stabilizing the foams are mainly the unbranched Gram positive filamentous bacterium “*Candidatus Microthrix parvicella*” and short Gram positive branching filamentous bacteria (the mycolata; Kragelund et al., 2007; Seviour et al., 2008; De Los Reyes, 2010). The latter includes members from the genera *Gordonia*, *Skermania*, *Rhodococcus*, *Nocardia*, *Tsukamurella*, and *Mycobacterium* (Seviour et al., 2008). Although improved culturing practices and molecular methods (such as 16S/23S rRNA targeted probes) have allowed the isolation and identification of numerous filamentous bacteria which are responsible for the formation of the stable foams (Speirs et al., 2009, 2011; Seviour and Nielsen, 2010), there are currently no reliable strategies to control sludge bulking during water treatment.

One attractive approach to preventing or eliminating sludge bulking and foaming caused by biological contaminants in treatment plants is to use phage-therapy (Thomas et al., 2002). The use of lytic phages that target the causative bacteria is specific, relatively inexpensive and environmentally-friendly. Most mycolata-specific phages that have been isolated can prevent foaming in pure cultures under laboratory conditions (Petrovski et al., 2011b,c,d, 2012). However, the isolation of phages for *Gordonia amarae* and *Gordonia defluvii*, two of the major foaming bacteria, has been unsuccessful. It is possible that these organisms have evolved sophisticated mechanisms to prevent successful infection, such as clustered regularly interspaced short palindromic repeats systems, however, this is yet to be elucidated. The phages isolated and described so far possess long non-contractile tails, characteristic of the *Siphoviridae* family, and have a broad host range for several mycolata genera responsible for foaming (Thomas et al., 2002).

Some wastewater treatment methods rely on the use of antimicrobial agents to control proliferation of bacteria however, this is not a reliable strategy due to the elimination of bacteria beneficial to wastewater treatment processes. Phage-therapy can therefore be used as an efficient complementary technique to control the proliferation of recalcitrant bacteria to prevent sludge bulking and foaming (Thomas et al., 2002; Withey et al., 2005). Although phage-therapy is effective in controlling the proliferation of wastewater bacteria in the laboratory, full-scale studies are required prior to the implementation of this approach in an industrial setting. Activated sludge systems in treatment plants are complex engineered ecosystems that support the growth of a wide diversity of microbes, which are poorly understood (Withey et al., 2005). Even less information is available on phage diversity and ecology in wastewater treatment systems. Phage survival rate in activated sludge has not been studied, and the ability of phages to infect their hosts may be inactivated or blocked by chemicals in wastewater. Furthermore, the host may evolve to develop resistance to the phage and a combination of phages will be required to tackle a large proportion of bacteria.

## Concluding Remarks

Our understanding of the structure and function of microbial communities in contaminated aquatic environments is allowing the manipulation of their innate biosensing and biodegradative abilities. Currently used physico-chemical methods to remove heavy metals are not effective at low concentrations but could be overcome with the use of biological approaches. The biological approaches described in this mini-review could serve as complementary techniques to chemical treatments and allow real-time measurements for prompt detection of contaminants (Figure 1) and treatment of contaminated water (Figure 2). The innovative approaches described can be used in conjunction with existing technologies to improve biosensing and biodegradation processes. In addition, the implementation of these approaches as an autonomous platform could allow for continuous *in situ* monitoring of contaminants at remote locations and could overcome some of the limitations of the current processes. Phage-therapy could be used to target pathogenic/detrimental bacterial species, thus serving as a cost-effective and environmentally-friendly approach to treatment of biologically-contaminated water.

### Conflict of Interest Statement

The authors declare that the research was conducted in the absence of any commercial or financial relationships that could be construed as a potential conflict of interest.
